# Meta-mining of copy number profiles of high-risk neuroblastoma tumors

**DOI:** 10.1038/sdata.2018.240

**Published:** 2018-10-30

**Authors:** Pauline Depuydt, Jan Koster, Valentina Boeva, Toby D. Hocking, Frank Speleman, Gudrun Schleiermacher, Katleen De Preter

**Affiliations:** 1 Center for Medical Genetics, Ghent University, Ghent, Belgium; 2 Cancer Research Institute Ghent (CRIG), Ghent University, Ghent, Belgium; 3 Department of Oncogenomics, Academic Medical Center, University of Amsterdam, Amsterdam, The Netherlands; 4 Institut Cochin, Inserm U1016, CNRS UMR 8104, Université Paris Descartes UMR-S1016, F-75014 Paris, France; 5 Institut Curie, Inserm U900, Mines ParisTech, PSL Research University, F-75005 Paris, France; 6 Department of Human Genetics, McGill University, Montreal, Quebec, Canada; 7 Recherche Translationelle en Oncologie Pédiatrique (RTOP) and Department of Pediatric Oncology, Institut Curie, Paris, France; 8 U830, INSERM, Paris, France

**Keywords:** Prognostic markers, Paediatric cancer, Translational research

## Abstract

Neuroblastoma, a pediatric tumor of the sympathetic nervous system, is predominantly driven by copy number aberrations, which predict survival outcome in global neuroblastoma cohorts and in low-risk cases. For high-risk patients there is still a need for better prognostic biomarkers. Via an international collaboration, we collected copy number profiles of 556 high-risk neuroblastomas generated on different array platforms. This manuscript describes the composition of the dataset, the methods used to process the data, including segmentation and aberration calling, and data validation. t-SNE analysis shows that samples cluster according to *MYCN* status, and shows a difference between array platforms. 97.3% of samples are characterized by the presence of segmental aberrations, in regions frequently affected in neuroblastoma. Focal aberrations affect genes known to be involved in neuroblastoma, such as *ALK* and *LIN28B*. To conclude, we compiled a unique large copy number dataset of high-risk neuroblastoma tumors, available via R2 and a Shiny web application. The availability of patient survival data allows to further investigate the prognostic value of copy number aberrations.

## Background & Summary

Neuroblastoma is a pediatric tumor of the sympathetic nervous system, affecting mainly children under the age of five years^1^. It is the most common extracranial solid cancer, making up 5% of childhood cancer diagnoses, while accounting for approximately 10% of childhood cancer deaths^2,3^.

Neuroblastoma is characterized by extensive clinical heterogeneity, illustrated by different clinical evolutions ranging from spontaneous regression to aggressive disease. Risk stratification of neuroblastoma patients is based on clinical parameters, histopathological parameters and genetic parameters including *MYCN* amplification, 11q loss and the global copy number profile^4,5^.

While recurrent single nucleotide mutations, such as those targeting *ALK* and *ATRX*, are observed infrequently at diagnosis^6,7^, copy number aberrations occur at much higher frequency and are strongly associated with disease outcome. Tumors with only numerical aberrations have a favorable prognosis, while the presence of at least one segmental aberration is indicative of poor survival outcome^8^.

Neuroblastoma is a rare disease, therefore it is challenging to collect enough tumor material and data for a sufficiently powered study. Within the Ultra-High-Risk (UHR) working group of the International Neuroblastoma Response Criteria (INRC) consortium^5^, we collected DNA copy number profiles and clinical data of 556 high-risk neuroblastoma patients, resulting in a unique dataset. The aim of this project was to explore whether specific (combinations of) segmental DNA copy number aberrations allow to better discriminate high-risk patients with fatal outcome from high-risk patients with favorable outcome^9^.

**Figure 1:**
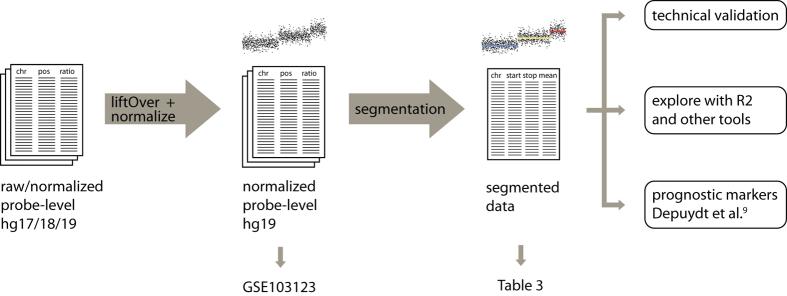
Overview of copy number data processing of 556 high-risk primary neuroblastoma tumors from probe-level to segmented data. Raw/normalized probe-level data were converted to hg19 genome build using liftOver, followed by median normalization and segmentation using SegAnnDB. Data are available in Gene Expression Omnibus (GEO). Segmented data are available in Table 3.

The collected copy number profiles were generated on several aCGH and SNP array platforms. The data were uniformized by converting to the same genome build hg19, and by segmenting with the same method (summarized in Fig. 1). From the segmented data, aberrations were called using specific cutoffs for each array platform. Exploration of the collected and processed data confirmed the presence of segmental and focal aberrations in regions frequently affected in neuroblastoma and that samples cluster according to *MYCN* status rather than platform and inter-laboratory differences.

In this data descriptor, we describe in detail the composition of the data with respect to sample inclusion criteria and clinical characteristics and the methods used to process the data. We provide access to normalized probe-level data and segmented data as well as instructions to analyze and visualize the data using either the R2 platform^10^, the statistical programming language R or a Shiny web application.

## Methods

### DNA copy number data collection

Copy number profiling data from 556 primary neuroblastoma tumors were collected through the UltraHigh-Risk (UHR) working group of the International Neuroblastoma Response Criteria (INRC) consortium. These tumors originated from high-risk patients enrolled in the SIOPEN, GPOH, COG or Japanese treatment protocol (SIOPEN: European Society for Paediatric Oncology Neuroblastoma, GPOH: Society for Paediatric Oncology and Haematology, COG: Children’s Oncology Group). Published^6,11,12^ and unpublished copy number profiling data generated on different platforms including aCGH arrays from Agilent (resolution ranging from 44k-1M) and NimbleGen (72k) and SNP arrays from Affymetrix (250k and 2.6 M) and Illumina (550k) were included in this study. Initially, data from 671 high-risk samples were collected, with high-risk defined as either stage 4 and older than 1 year, or *MYCN*-amplified (any age and stage). *MYCN* amplification status was determined by FISH or qPCR. Of the 671 samples, 16 samples were discarded due to bad quality profiles and 98 samples were discarded due to suspected contamination with normal cells (49 silent profiles without detected copy number aberrations and 50 with almost undetectable aberrations), resulting in a set of 556 samples. Sample annotations, including among others age at diagnosis, disease stage, *MYCN* status and overall and event-free survival, are available at Figshare (Table 1: Patient annotations, Data Citation 1). However, event-free survival data are not available for the Japanese dataset. This collection strategy has been reported already in the Supplementary Methods of the research paper originally related to this dataset^9^. The patient annotations in this publication are the same as Supplementary Table 1 in the related paper^9^.

### DNA copy number data processing

The processing steps performed on the collected data to obtain segmented copy number data is summarized in Fig. 1. Raw or normalized copy number data were collected. Raw data were median-normalized. When necessary, chromosomal coordinates were converted from genome build hg17 or hg18 to genome build hg19 using the “liftOver” function from the R package “rtracklayer”. Only for the COG cohort (Illumina platform), instead of using liftOver, we replaced the probe positions with hg19 positions downloaded from the Illumina website. These normalized, hg19 probe-level copy number data are available at GEO with accession number GSE103123 (Data Citation 2). An overview of the processing steps applied to each of the collected datasets to obtain the data available at GEO is available at Figshare (Table 2: data processing steps, Data Citation 1). Starting from the normalized hg19 data, segmentation of the copy number profiles was done using SegAnnDB^13^ (http://seganndb.genap.ca/, a video with instructions can be found on the website). This is an interactive web-based tool that combines mathematical modelling with visual annotation to call aberrations, ensuring qualitative breakpoint calling for the majority of samples. Aberrations that were abundantly present across samples profiled on a certain platform were considered as artefacts (usually also a high variability in probe copy number value was observed for these regions) and ignored in the segmentation process. Due to the semi-automatic nature, not all aberrations are detected by this tool, e.g. subclonal aberrations might be missed. Only copy number data were used as input for the segmentation as allele ratios were available only for a limited number of samples. As not for all copy number profiling experiments gender-matched controls were used, data from chromosome Y were omitted. The segmented copy number profiles were downloaded from the SegAnnDB server for further analysis and are available at Figshare (Table 3: Segmented copy number data, Data Citation 1). A shorter version of these processing methods are included in the (Supplementary) Methods section of the related paper^9^.

### Defining cutoffs to call aberrations

**Figure 2:**
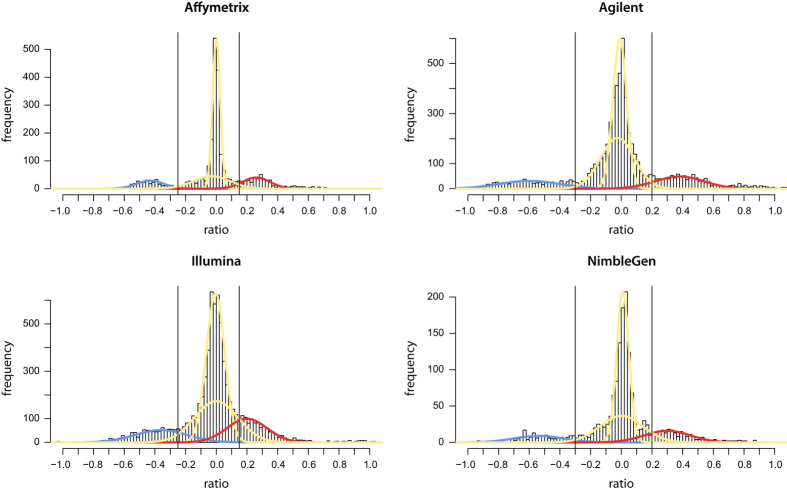
Histogram of log2 ratios of segments from all samples, according to platform. Cutoffs are determined based on intersecting yellow curves (normal segments) with blue or red curves (lost/gained segments). For SNP arrays (left) lower cutoffs are used compared to aCGH platforms (right) to call copy number aberrations. Figure adapted from Supplementary Figure 1 in related paper^9^.

**Figure 3:**
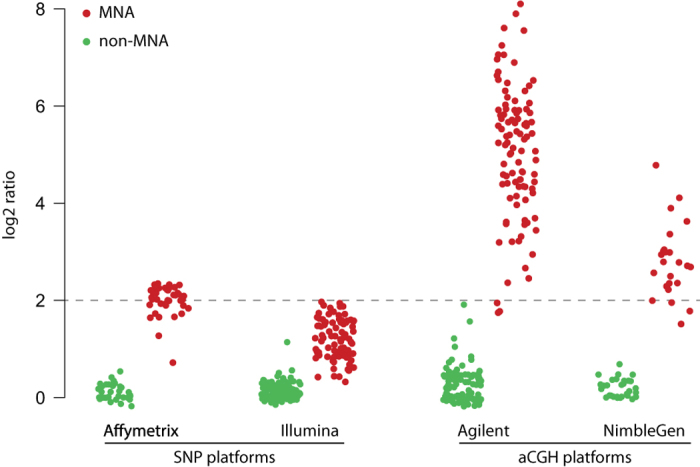
Comparison of *MYCN* copy number log2 ratios in *MYCN*-amplified and non-amplified cases, according to platform. *MYCN* log2 ratios of *MYCN*-amplified cases are lower for SNP arrays than aCGH arrays, justifying platform-specific cutoffs to call amplifications. Figure adapted from Supplementary Figure 10 in related paper^9^.

Histograms of the segment log2 ratios for each array platform reveal that their distribution is dependent on the array platform. More specifically, aCGH arrays generate log2 ratios with a higher dynamic range than SNP arrays (Fig. 2). Based on this observation, gains and losses were called using platform-specific cutoffs. Following cutoffs were used for gains/losses respectively: +0.15/-0.25 for SNP arrays (Affymetrix and Illumina), and +0.2/-0.3 for aCGH arrays (Agilent and NimbleGen), represented by the vertical lines in Fig. 2. Also for calling of amplifications platform-specific cutoffs were used. We compared copy number values (log2 ratio) of segments encompassing *MYCN* for *MYCN*-amplified and nonamplified samples (as determined based on FISH/qPCR analyses) (Fig. 3). Cutoffs were chosen to maximize discrimination between *MYCN*-amplified and nonamplified samples and exclude high-level gains: 1.5 for Affymetrix and NimbleGen arrays, 2 for Agilent arrays and 0.7 for Illumina arrays. Finally, segments with log2 ratio lower than -2 were called as homozygous deletion. This computational method using cutoffs generates a good general view of the aberrations present in the study population, but occasionally misses some aberrations, e.g. subclonal aberrations will typically not reach the cutoff. The resulting annotations are included in the segmented data available at Figshare (Table 3: Segmented copy number data, Data Citation 1). This paragraph is a more elaborate version of the procedure described in the related paper^9^.

### Presence of segmental aberrations of frequently affected chromosome arms

For each patient, we determined the presence of aberrations in any of the frequently affected chromosome arms, namely loss of 1p, 3p, 4p, 11q and 14q, and gain of 1q, 2p and 17q. Aberrations of (partial) chromosome arms were defined as gains and losses larger than 3 Mb, hereby excluding whole chromosome aberrations and amplifications. In case of aberrations spanning the centromere, the aberration is considered to be on the p arm if the part on p is longer and vice versa. Note that this is a computational scoring generating a general image of abundance of aberrations, not aiming to establish a detailed genomic profile for individual patients. The resulting annotations are included in the patient annotations available at Figshare (Table 1: Patient annotations, Data Citation 1). This procedure is also described in the Supplementary Methods of the related paper^9^.

### Code availability

The code to analyze or visualize the data in R can be found on GitHub (https://github.com/padpuydt/copynumber_HR_NB/). See Usage Notes for more information.

## Data Records

All tables are available at figshare (Data Citation 1). Clinical and genomic information of the patients (Table 1: Patient annotations, Data Citation 1) include age at diagnosis, disease stage, and overall and event-free survival, *MYCN* status and the presence of segmental aberrations of chromosome arms frequently affected in neuroblastoma, such as 1p loss and 2p gain. Normalized probe-level copy number data using genome build hg19 can be accessed through GEO, with accession number GSE103123 (Data Citation 2). An overview is provided of the processing steps that were performed on the collected data to obtain the data that is available on GEO (Table 2: data processing steps, Data Citation 1), as well as segmented copy number data (Table 3: Segmented copy number data, Data Citation 1), in which the column “annotation” reports on the presence of an aberration considering the platform-specific cutoffs (see Methods).

## Technical Validation

### Bias

**Figure 4:**
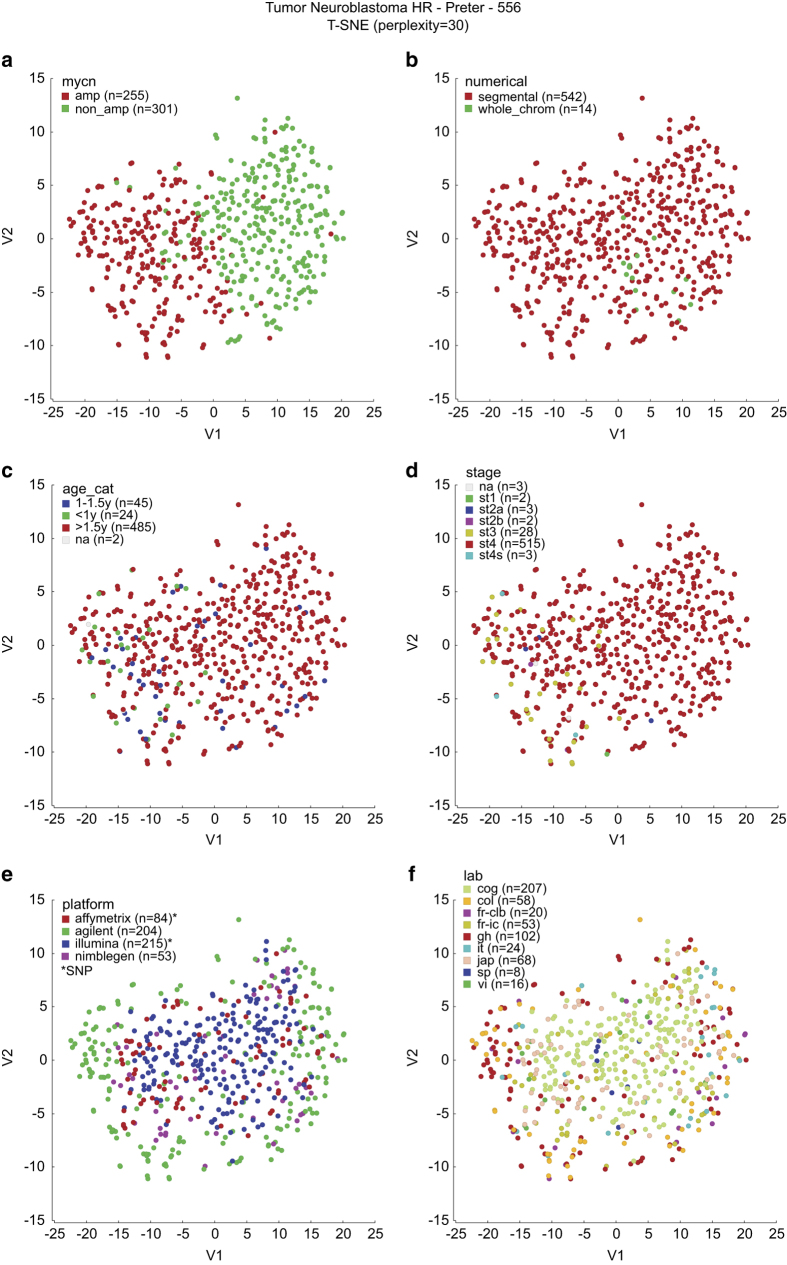
t-SNE analysis illustrating clustering of samples. Samples are colored according to genomic (top), clinical (middle) and technical (bottom) variables, indicating that technical bias is limited to variable dynamic ranges among platforms and that samples cluster according to *MYCN* status. cog: Children’s Oncology Group, USA; fr-clb: Centre Léon Bérard, France; fr-ic: Institut Curie, France; gh: Ghent, Belgium; it: Genova, Italy; jap: Chiba, Japan; sp: Valencia, Spain; col: Cologne, Germany; vi: Vienna, Austria.

To check for possible bias, we performed t-SNE analysis using the R2 platform^10^, for several genomic (top), clinical (middle) and technical (bottom) variables (Fig. 4). This analysis reveals a clear clustering of samples according to *MYCN* status (Fig. 4a). Samples with only numerical aberrations are somewhat clustering together and situated in between the *MYCN*-amplified and *MYCN*-nonamplified group (Fig. 4b). Samples slightly cluster according to the clinical variables age at diagnosis (in three categories) and stage of the disease, but this mainly reflects the fact that young ( < 1 year) and non-stage 4 patients were only included in the study when presenting with *MYCN* amplification (Fig. 4c and d). In Fig. 4e, a slight bias is observed for different array platforms, with samples analyzed on CGH arrays (especially Agilent) being on the outer rim, and samples analyzed on SNP arrays (especially Illumina) situated closer to the center. This can be explained by the fact that CGH arrays generate copy number log2 ratios with a higher dynamic range than SNP arrays, as also observed in the histograms in Fig. 2. This effect is demonstrated in the simulations by Wattenberg *et al.* (section 6)^14^ . In Fig. 4f, we demonstrate that there is no bias across labs that use the same platform.

### Detection of typical segmental and focal aberrations

**Figure 5:**
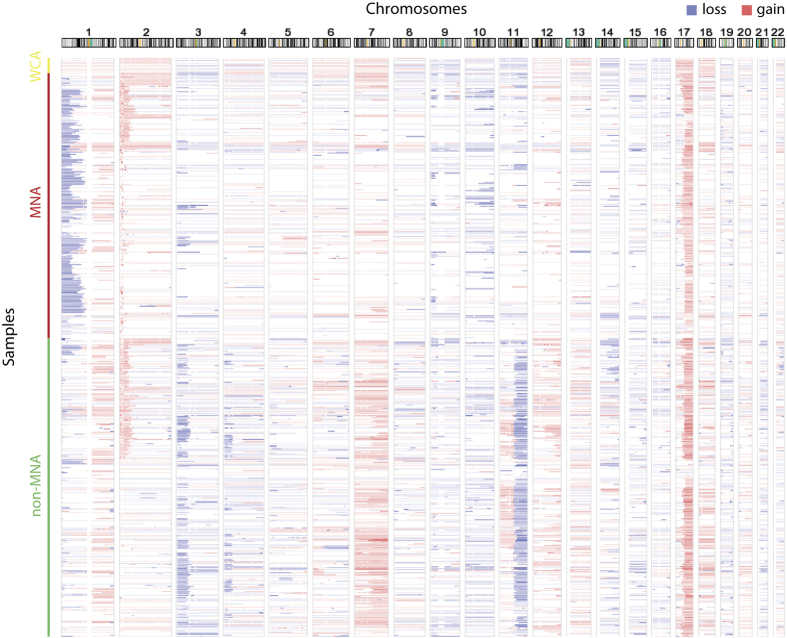
Copy number aberrations in all samples. Heatmap of aberrations in each individual sample, illustrating patterns of co-occurring aberrations, e.g. 11q and 3p loss, or *MYCN* amplification (MNA) and 1p deletion. WCA: whole-chromosome aberrations. Figure adapted from Supplementary Figure 5 in related paper^9^.

Of the 556 collected high-risk samples, 97.3% contain segmental aberrations, corresponding to regions frequently affected in neuroblastoma. The heatmap of chromosomal gains and losses of all tumors (Fig. 5) clearly shows recurrent loss of 1p, 3p, 4p, and 11q and recurrent gain of 1q, 2p, 7 and 17q. This visualization confirms previous reports^15^ that most tumors with *MYCN* amplification also have 1p loss and 17q gain and that many tumors with 11q loss also present with 3p loss and 17q gain. A frequency plot of the gains and losses can be found in Fig. 1b of the related research paper^9^. Next to larger segmental aberrations, also focal (<5 Mb) aberrations were observed that have previously been described in neuroblastoma, including focal gains or amplifications of *ALK* and *LIN28B*.

### Detection of amplifications

In order to set a good cut-off to determine amplifications, log2 copy number ratios of the *MYCN* locus were compared in samples with or without *MYCN* amplification according to FISH and qPCR. This analysis (Fig. 3) reveals that the dynamic range of log2 ratios is highly variable between platforms and suggests that cutoffs used to call amplifications should be determined per platform. Using these cutoffs we can correctly determine *MYCN* amplification status (as previously established by FISH/qPCR) in 97% of Affymetrix samples, 95% of Illumina samples, 99% of Agilent samples and 100% of NimbleGen samples. As described in the related paper^9^ and by Curtis *et al.*^16^, detection of amplifications is more cumbersome in data from Illumina platforms and should be interpreted with caution.

## Usage Notes

Probe-level data can be accessed at GEO with the accession number GSE103123 (Data Citation 2). Sample annotations are provided in the GEO series metadata and at Figshare (Table 1: Patient annotations, Data Citation 1). Instead of downloading probe-level data, it is also possible to directly use the segmented data, also available at Figshare (Table 3: Segmented copy number data, Data Citation 1). The segmented profiles have been manually inspected to ensure confidence of the called aberrations. The column “annotation” represents the presence of an aberration considering the platform-specific cutoffs (see Methods for details), but also the segment log2 ratios can be used.

Data can be analyzed and visualized using either the R statistical program, the R2 web-based platform^10^, or a Shiny web application^17^. For R users we recommend the R package “copynumber”^18^, which provides functions for visualization, including frequency plots and heatmaps with chromosome ideograms. The R package CNTools^19^ can be used to transform the segmented data into a window- or gene-based matrix. A script is provided on GitHub, including functions to select samples with an aberration for a certain gene/region (https://github.com/padpuydt/copynumber_HR_NB/).

**Figure 6:**
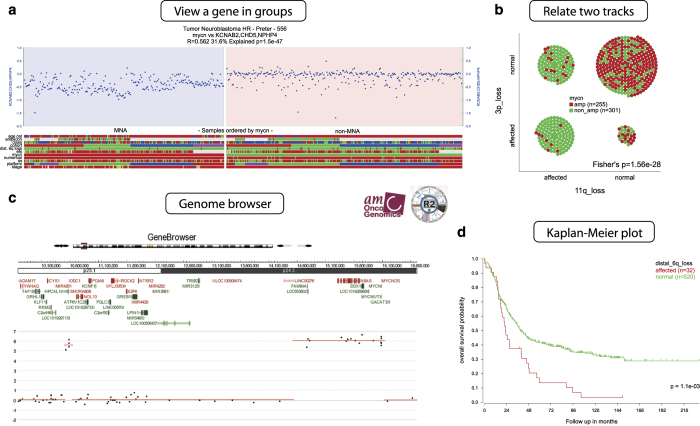
R2 platform: examples of data exploration and visualization. (**a**) Copy number values of *CHD5* (1p36.31), comparing samples with *MYCN* amplification (left) and without *MYCN* amplification (right). Other sample annotations including age at diagnosis can be read in the heatmap below the plot. (**b**) Comparing occurrence of two tracks, illustrating that 11q and 3p loss significantly co-occur. (**c**) Genome browser zooming in on a sample with amplifications for *ODC1* (left amplified region) and *MYCN* (right amplified region). (**d**) Kaplan-Meier analysis comparing survival of cases with and without 6q loss.

Using the R2 platform^10^, one can perform multiple data analysis procedures and create several types of plots. A few examples are plotting a gene’s copy number in groups of samples, associations between sample annotations, survival analysis and viewing samples in the genome browser (Fig. 6). To create these plots, go to the dataset home page on R2 (https://hgserver1.amc.nl/cgi-bin/r2/main.cgi?&dscope=NB556HR&option=about_dscope). The R2 genome browser is directly linked there. The other three analyses/plots can be found under “Other Analyses”. There we selected “View a gene in groups”, “Relate 2 tracks” and “Kaplan Meier by annotated parameter” in step 3 (Select type of analysis). In addition, R2 allows to perform correlation analysis between genes, find genes with differential copy number between groups, perform dimensionality reduction with t-SNE maps, or generate a heatmap of a specific (grouped) subset of samples (CGH Sample Sorter on dataset home page). More detailed documentation can be found on the R2 website (http://r2.amc.nl).

Finally, we developed a Shiny web application^17^ to interrogate the copy number status and association with survival of any gene or genomic region of interest. After filling in the gene name or cytoband, the samples with an aberration overlapping with that region will be plotted in a heatmap. One can also plot survival curves of samples with versus without an aberration in the region of interest. A preliminary version can be accessed through https://padpuydt.shinyapps.io/check_cn_in_hr_nb/.

## Additional information

**How to cite this article**: Depuydt. P. *et al*. Meta-mining of copy number profiles of high-risk neuroblastoma tumors. *Sci. Data*. 5:180240 doi: 10.1038/sdata.2018.240 (2018).

**Publisher’s note**: Springer Nature remains neutral with regard to jurisdictional claims in published maps and institutional affiliations.

## Supplementary Material
